# A Fatal Case of Life-Threatening Interstitial Pneumonitis Induced by Everolimus for Metastatic Renal Cell Carcinoma: A Comment about the Increased Risk of Interstitial Lung Disease in Japanese

**DOI:** 10.1155/2019/2703871

**Published:** 2019-01-31

**Authors:** Yasukiyo Murakami, Tetsuo Fujita, Yoji Wakatabe, Masatsugu Iwamura

**Affiliations:** Department of Urology, Kitasato University School of Medicine, Japan

## Abstract

We describe an 81-year-old woman with metastatic renal cell carcinoma who did not recover from life-threatening interstitial pneumonitis induced by everolimus therapy. She received everolimus due to disease progression after sunitinib, but 2 months after starting everolimus treatment, she visited the emergency department after developing a sudden fever and dyspnea. Chest CT revealed diffuse ground-glass opacities, thickening of the interlobular septa, and consolidation throughout both lung fields. The diagnosis was surmised to be everolimus-induced interstitial pneumonitis. Everolimus administration was stopped and 3 courses of steroid pulse therapy were administered, along with intensive care, but the patient died due to rapid respiratory failure.

## 1. Introduction

The mTOR (mammalian target of rapamycin) inhibitors for the treatment of advanced renal cell carcinoma have expanded the available treatment options with molecular-targeted drug therapies. Among mTOR inhibitors, everolimus is positioned as a sequential therapy agent [1]. Interstitial lung disease (ILD) is listed as an adverse event that requires caution, but severe ILD resistant to appropriate treatment is rare. We observed a case of life-threatening interstitial pneumonitis induced by everolimus in a patient with metastatic renal cell carcinoma and reported our findings.

## 2. Case Presentation

**Figure 1:**
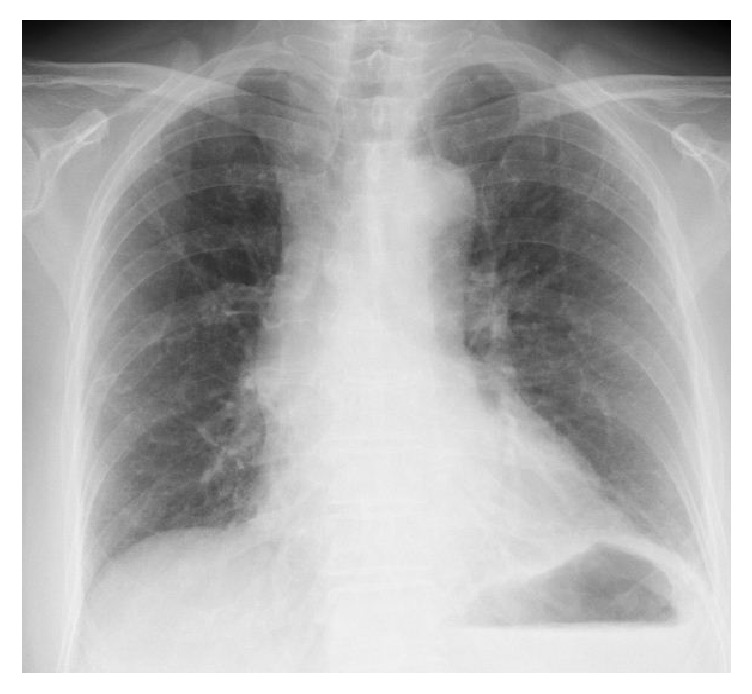
Chest X-ray one and a half months after the initiation of everolimus treatment, showing no infiltrative shadows in both lung fields.

**Figure 2:**
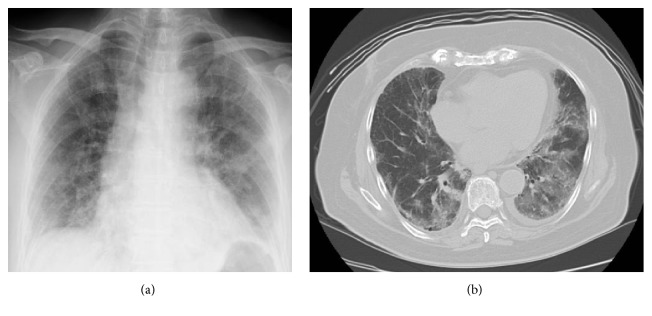
(a) Chest X-ray two months after starting everolimus administration, showing diffuse infiltrative shadows in both lung fields. (b) Chest CT scan two months after starting everolimus administration, showing ground-glass attenuation with diffuse alveolar consolidation in both lung fields.

The patient was an 81-year-old woman with metastatic renal cell carcinoma. Due to a clinical suspicion of renal cell carcinoma, patient initially underwent right radical nephrectomy. The histopathological diagnosis was pT2 clear cell carcinoma. After a 10-year disease-free interval, distal pancreatectomy and splenectomy were performed for pancreatic mass lesion recurrence. Two years later, recurrence at the site of the pancreatectomy was diagnosed by an abdominal CT scan, and then further surgical resection of the recurrent tumor was performed. However, a recurrent mass lesion was found at the head of the pancreas a year after surgical resection of the recurrent tumor. As surgical resection was not indication for treatment due to postoperative adhesions, sorafenib (800 mg/day) was initiated. The lesion persisted in a stable disease condition, but 16 months after starting the sorafenib therapy, the metastatic lesion at the head of pancreas became a progressive disease, so the regimen was switched to sunitinib (37.5 mg/day). However, 4 months later, a CT scan showed disease progression with the appearance of liver metastatic lesions, so everolimus (10 mg/day) was initiated. In evaluation prior to everolimus, there were no findings of respiratory dysfunction. Arterial blood gas analysis revealed a pH of 7.333, PaCO2 40.0* *mmHg, bicarbonate 20.8 mmol/L, and PaO2 10.5 mmHg on 97.5% FiO2. Laboratory data also showed normal CRP levels. No apparent changes, including interstitial opacities, were observed on the chest CT taken 1 month after starting everolimus administration. At one and a half months after everolimus induction, the patient showed no remarkable respiratory symptom and no remarkable change was seen in the patient's chest X-ray (Figure 1). Two months after starting everolimus administration, the patient presented to the emergency department after developing a sudden fever and dyspnea. Her peripheral capillary oxygen saturation level was 93% (under inhalation of O2 3 L), and blood gas analysis revealed decompensated alkalosis. The results of the general blood biochemistry tests were normal apart from an elevated C-reactive protein level of 13.93 mg/dl. Her blood serum KL6 level was elevated at 1929 IU/ml, as was her surfactant protein A (SP-A) and surfactant protein D (SP-D) levels, to 103.0 and 513.0 IU/ml, respectively. Her serum *β*D-glucan level was within the normal range. Linear, reticular shadows were found in both lung fields during chest radiography (Figure 2(a)), and a chest CT revealed diffuse ground-glass opacities, thickening of the interlobular septa, and consolidation throughout both lung fields. Mild pericardial effusion was found, but there is no findings of suspecting cardiogenic pulmonary edema, which indicate acute respiratory distress syndrome (Figure 2(b)). The diagnosis was surmised to be everolimus-induced interstitial pneumonitis. The patient was immediately treated with oxygen and steroid pulse therapies (methylprednisolone 1 g/day for 3 days) by a respiratory specialist, and everolimus administration was promptly stopped. The patient's respiratory status continued to rapidly worsen, however. The patient received ventilation on day 3 of hospitalization at the intensive care unit. The possibility of pneumonitis caused by infection, including fungal infection, was ruled out after subsequent culture tests returned negative. Accordingly, a respiratory specialist concluded the diagnosis as everolimus-induced interstitial lung disease. The patient had two more courses of steroid pulse therapy but showed no improvement in her respiratory status. The patient died on day 49 of hospitalization due to rapid respiratory failure.

## 3. Discussion

Everolimus is an oral mTOR inhibitor widely used for metastatic renal cell carcinoma, which is presented as a subsequent therapy option in accordance with the National Comprehensive Cancer Network guidelines [1]. Noninfectious pneumonitis, including ILD, is one of the most important adverse events that require attention during everolimus treatment. This adverse event is considered a class effect of rapamycin derivatives [2].

Reports indicate that the incidence of noninfectious pneumonitis with the use of everolimus ranges from 13.5% to 27% [3–6]. In an international randomized phase III RECORD1 trial for the treatment of metastatic renal cancer, noninfectious pneumonitis developed in 14% of all patients, while grade 3/4 complications occurred in 4% of patients [6]. Another blinded prospective study including 274 patients with metastatic renal cancer who received everolimus found that clinical noninfectious pneumonitis was suspected for 13.5% patients, but no life-threatening severe pneumonitis was observed [7]. It is reported that the incidence of this type of pneumonitis has a racial difference and Japanese patients have a higher incidence of noninfectious pneumonitis [8, 9]. In the Japanese subgroup analysis of the RECORD-1 Trial, the prevalence of noninfectious pneumonitis was higher than in the overall population (Japanese vs. overall: 27% vs. 13.5%, respectively) [10]. The reason for the higher incidence of noninfectious pneumonitis in Japanese patients is still not fully understood. However, differences in genetic sensitivity may have a role in the adverse reaction [11]. Indeed, in the treatment of nonsmall cell lung cancer, Japanese patients are known to have a higher incidence of severe drug-induced ILD caused by the tyrosine kinase inhibitor gefitinib [12]. Individual cancer type and aggressiveness may also affect the incidence of adverse reactions in addition to racial difference, because the incidence of respiratory-related adverse events has been reported to be extremely low in patients with renal angiomyolipoma treated with everolimus therapy [5]. These results suggest that, other than race, identifying the risk factors for drug-induced ILD will help prevent disease development. During gefitinib administration, preexisting pulmonary fibrosis with a performance status ≥2 has been identified as a risk factor for developing ILD in Japanese cohort [13]. However, the risk factors for everolimus-induced ILD are not detected due to the small sample size (Table 1). Therefore, close respiratory follow-up will be needed for patients with renal cell carcinoma using Everolimus. Moreover, further multiinstitutional investigation is warranted to determine the risk factor of everolimus-induced ILD.

It is recommended that treatment for everolimus-induced ILD is in line with the severity of the condition [17]. If the radiological findings are positive in an otherwise asymptomatic condition, treatment could be continued without cessation of everolimus therapy, because even severe cases respond well to steroid therapy [18]. The respiratory status of the present patient did not improve even after 3 courses of steroid pulse therapy, and the patient ultimately died from respiratory failure caused by ILD. As far as we could find, this is the relatively rare reported case of fatal everolimus-induced ILD. KL-6, surfactant protein (SP)-A, SP-D, are reported to be sensitive markers for interstitial lung diseases (ILD) [16]. Of these markers, KL-6 is useful for making a differential diagnosis between interstitial pneumonia and other kinds of pneumonia. KL-6 is a mucinous high-molecular weight glycoprotein that is expressed on type II pneumocytes, which is highly sensitive and specific and correlates with the ground-glass opacities on the chest CT. This marker is also considered useful for detecting everolimus-induced ILD because KL-6 demonstrated a stronger correlation with fibrotic lesions than the other markers [19]. In a report that investigated 7 patients with metastatic renal cell carcinoma who developed ILD after administration of everolimus, all of the patients had an elevated KL-6 level [3]. In the current case, the KL-6 level increased to 1929 IU/ml immediately after the onset of ILD and the KL-6 levels continued to increase despite steroid therapy, reaching 2490 IU/ml immediately before the patient died. KL-6 is also recognized as being useful for assessing the disease activity of interstitial pneumonia. Reports on idiopathic ILD indicated that patients with ILD who had KL-6 levels of ≥1000 IU/ml had a poorer prognosis than those with KL-6 levels of <1000 IU/ml [20]. This study suggests the potential usefulness of KL-6 as a prognostic factor.

Everolimus-induced ILD is manageable in almost cases, but our case suggests that ILD during everolimus treatment still requires attention. Further investigation on follow-up examinations of KL-6 levels and everolimus-induced ILD prognostic factors is necessary.

## 4. Conclusion

We observed a case of severe interstitial pneumonitis in a patient who was receiving everolimus therapy for advanced renal cell carcinoma that resulted in a serious outcome, and we reported our findings along with the potential to use KL-6 levels during follow-up examinations as prognostic factor for everolimus-induced ILD.

## Figures and Tables

**Table 1:** Comparison of previous studies regarding interstitial lung disease associated with TKI or mTOR inhibitor in Japanese patients with renal cell carcinoma and lung cancer.

				% of ILD		
Series	Type of cancer	Type of drug	n	All grades	Grade 3/4	Grade 5
Mizuno et al. [3]	RCC	mTOR inhibitor (Everolimus)	11	63.6	36.3	0
Mikami et al. [14]	RCC	mTOR inhibitor (Everolimus)	16	31	0	0
Akamatsu et al. [15]	NSCLC	TKI (Gefetinib)	201	5	2.5	1
Nakagawa et al. [16]	NSCLC	TKI (Erlotinib)	3488	4.5	2.6	1.6
Ando et al. [13]	NSCLC	TKI (Gefetinib)	1976	3.5	1.9	1.6

RCC, renal cell carcinoma; NSCLC, nonsmall cell lung carcinoma; ILD, interstitial lung disease; mTOR, mammalian target of rapamycin; TKI, tyrosine kinase inhibitor.
